# Enlarged extracellular vesicles are a negative prognostic factor in patients undergoing TACE for primary or secondary liver cancer–a case series

**DOI:** 10.1371/journal.pone.0255983

**Published:** 2021-08-18

**Authors:** David Schöler, Mirco Castoldi, Markus S. Jördens, Max Schulze-Hagen, Christiane Kuhl, Verena Keitel, Tom Luedde, Christoph Roderburg, Sven H. Loosen

**Affiliations:** 1 Clinic for Gastroenterology, Hepatology and Infectious Diseases, University Hospital Düsseldorf, Medical Faculty of Heinrich Heine University Düsseldorf, Düsseldorf, Germany; 2 Department of Diagnostic and Interventional Radiology, University Hospital RWTH Aachen, Aachen, Germany; University of Navarra School of Medicine and Center for Applied Medical Research (CIMA), SPAIN

## Abstract

**Background:**

Transarterial chemoembolization (TACE) has evolved as a standard treatment option in patients with intermediate stage, unresectable HCC [Barcelona Clinic Liver Cancer (BCLC) stage B] as well as in patients with liver metastases, when surgery or systemic therapy is considered not appropriate. Concentration and sizes of extracellular vesicles (EVs) recently emerged as novel diagnostic and prognostic biomarkers in patients with liver cancer, but no data on its prognostic relevance in the context of TACE exists. Here, we evaluate pre-interventional EVs as a potential biomarker in patients undergoing TACE for primary and secondary hepatic malignancies.

**Methods:**

Vesicle size distribution and concentration were measured by nanoparticle tracking analysis (NTA) in patient sera before and after TACE in 38 patients.

**Results:**

Extracellular vesicle size distribution measured before TACE is of prognostic significance with respect to overall survival in patients after TACE. Overall survival is significantly reduced when initial vesicle size (X50) is in the upper quartile (>145.65nm). Median overall survival in patients in the upper quartile was only 314 days, compared to 799 days in patients with vesicle size in the first to third quartile (<145.65nm; p = 0.007). Vesicle size was also shown to be a significant prognostic marker for overall survival in Cox regression analysis [HR 1.089, 95% CI: 1.021–1.162, p = 0.010]. In addition, a significant correlation was observed between initial EVs concentration/BMI (r_S_ = 0.358, p = 0.029), X50/IL-8-concentration (r_S_ = 0.409, p = 0.011) and X50/CRP-concentration (r_S_ = 0.404, p = 0.016). In contrast, with regard to immediate tumor response after TACE, EVs concentration and size did not differ.

**Summary:**

Sizes (but not concentrations) of EVs represent a novel prognostic marker in patients receiving TACE for primary and secondary hepatic malignancies since patients with enlarged EVs display a significantly impaired prognosis after TACE.

## Introduction

Liver tumors represent an enormous medical and socioeconomic problem worldwide. In addition to primary liver tumors such as hepatocellular carcinoma, liver metastases from other tumors are responsible for nearly 90% of all malignant liver lesions [1]. With the introduction of new multimodal therapeutic strategies, the prognosis of patients with both primary and secondary liver tumors has improved significantly [2, 3]. In particular, the introduction of so-called local ablative and intra-arterial therapeutic procedures has revolutionized the clinical management of patients with liver tumors [2, 3]. Most importantly, transarterial chemoembolization (TACE) has evolved as a standard treatment option providing an acceptable balance between anti-tumor effect and toxicity both in patients with intermediate stage, unresectable HCC [Barcelona Clinic Liver Cancer (BCLC) stage B] as well as in CRC-patients, when surgery or systemic therapy is considered not appropriate [4, 5]. However, response rates after TACE therapy are highly variable and clinical benefit varies considerably between different patients. Pre-interventional stratification for optimal patient selection remains therefore represent an important clinical need in the context of TACE [6, 7].

Extracellular vesicles (EVs) form a heterogeneous group of membrane-bound vesicles that contain cell-derived biomolecules, such as proteins, lipids, cytokines hormones as well as genetic material in the form of DNA, mRNAs and non-coding RNAs [8]. EVs might be released into the blood by many different cell types including malignant cells [8]. Extracellular vesicles are classified into several subtypes, each with different mechanisms of biogenesis and potentially reflecting different physiological and/ or pathophysiological processes [8]. Just recently concentrations and sizes of EVs emerged as biomarkers in the context of inflammatory and malignant diseases [9, 10]. As an example, alterations of total serum extracellular vesicle concentrations were found in HCC allowing to discriminate patients with early HCC from patients with liver cirrhosis only [11, 12].

In the present study, we aimed at evaluating concentrations and sizes of circulating EVs as novel biomarker for prediction of response and prognosis in patients undergoing TACE for primary and secondary liver cancer.

## Patients and methods

### Study design

This exploratory observational cohort study aims at evaluating a previously unrecognized role of extracellular vesicles as a novel biomarker in liver cancer patients receiving TACE therapy. A total of 38 patients diagnosed with primary or secondary liver cancer (HCC: n = 27, liver metastasis: n = 9) admitted to the Department of Medicine III who received TACE at the Department of Diagnostic and Interventional Radiology at University Hospital RWTH Aachen were enrolled between 2013 and 2017. Blood samples were drawn at the day before TACE and one day after TACE. Blood samples were centrifuged for 10 min at 2000 G and aliquots of serum were stored at −80°C until analysis. Ethical approval was granted by the ethics committee of the University Hospital RWTH Aachen, Germany (EK 206/09). The study was conducted in accordance with the standards of the Declaration of Helsinki. Written informed consent was obtained from patients.

### Measurement of extracellular vesicle concentration and size

Analysis of EVs was performed with ZetaView multi parameter Particle Tracking Analyzer (ParticleMetrix, Germany) to measure the quantity and size distribution of vesicles. The ZetaView determines the size of vesicles based on Brownian motion and this principle is used for analysis of nanometer-sized particles [13, 14]. Before measurements, accuracy of the ZetaView was assessed by performing auto-alignment by using a standard calibration nanoparticle solution provided by ParticleMetrix (diameter 110 nm). Before recordings, camera focus was adjusted to make the particles to appear as sharp dots. The sample expected to encompass the highest vesicle number was used to set the camera sensitivity, which was kept constant for the following measurements. Samples were diluted in particle-free PBS to achieve a particle count in the range of 1–9 x 10^7^ p./mL (or about 200 particles per visual field, PVF). Using the script control function, three 30-second videos for each sample were recorded, incorporating a sample advance and a 5-second delay between each recording.

### Transarterial chemoembolization (TACE)

Primary and secondary liver cancer was treated using an emulsion of a chemotherapeutic agent as well as an embolic agent that was diluted with iodized contrast (Ultravist 300, Bayer Vital GmbH, Leverkusen, Germany). HCC patients were treated with Doxorubicine and ethiodized oil (Lipiodol, Guerbet LLC, Bloomington, IN, USA). Intrahepatic metastases from other solid tumor entities including gastric, colorectal or pancreatic cancer were treated with a chemotherapeutic agent according to individual guideline and Lipiodol, degradable starch microspheres (EmboCept S, PharmaCept GmbH, Berlin, Germany) or drug eluting beads (DcBeads, BTG International Ltd, London, UK). All TACE procedures were conducted through the right femoral artery. Both a hepatography and a contrast-enhanced cone-beam CT in late arterial contrast phase were performed using a 2.4/2.7F-microcatheter. We performed a superselective (subsegmental), selective (segmental) or non-selective (lobar) approach depending on the entity, number, size, localization as well as arterial supply of the tumor.

### Determination of tumour response following TACE therapy

All study patients underwent a multidetector CT with multiphasic, contrast-enhanced acquisitions in native, arterial, portal venous and late-venous phase or a multiphasic, contrast enhanced liver MRI (1,5T, Philips Medical Systems DMC GmbH, Hamburg, Germany) not earlier than four weeks prior and approximately four weeks after TACE to individually assess the tumor response to TACE therapy. The CT/MRI scans were evaluated based on RECIST 1.1 criteria (non-arterially enhanced tumor entities) [15] or mRECIST criteria (HCC) [4]. Tumor response was categorized based on standard nomenclature for RECIST1.1/mRECIST: Complete response (CR), partial response (PR), stable disease (SD) and progressive disease (PD). CR and PR were classified as objective response (OR) [16].

### Statistical analysis

Statistical analyses were performed as recently described in detail [17]. Shapiro-Wilk-Test was performed to test for normal distribution. Non-parametric data were compared by Mann-Whitney-U-Test as well as Kruskal-Wallis-Test. Related samples were compared using Wilcoxon signed-rank test. Box plot graphics show the median, quartiles and ranges. Kaplan-Meier curves display the impact of EVS concentration or size on overall survival (OS). Log-rank test was used to test for statistical differences. The prognostic value of EVS concentrations and size were also tested by uni- and multivariate Cox regression analyses. Parameters with a p-value of <0.250 in univariate testing were included into multivariate testing. The hazard ratio (HR) and the 95% confidence interval are displayed. All statistical analyses were performed with SPSS 23 (SPSS, Chicago, IL, USA). A p-value of < 0.05 was considered statistically significant (* p < 0.05; ** p < 0.01; *** p < 0.001).

## Results

### Characteristics of study cohort

In the present analysis, 38 patients with primary (HCC) or secondary liver (liver metastases) cancer scheduled to receive TACE therapy were included. 76.3% of liver cancer patients were male and 23.7% were female. The cohorts’ median age was 66.5 years and ranged from 37–89 years. 76.3% of patients presented with HCC and 23.7% of patients had been diagnosed with hepatic tumor spreading from other non-hepatic malignancies. Among these, colorectal carcinoma was the most prevalent primary tumor site (10.5%). The median size of the TACE target lesion was 2.8cm and ranged from 1.2 to 12.9 cm. Among HCC patients with diagnosed liver cirrhosis, chronic alcohol intake (31.0%) and HCV infection (20.7%) were the most prevalent underlying disease etiologies. 45.2% of patients had an objective response (OR) to TACE and 54.8% were classified as non-OR. During follow-up 73% of patients deceased. Table 1 provides detailed characteristics of the study population.

**Table 1:** Characteristics of study cohort.

	Study cohort
TACE Patients [n]	38
Sex [%]	
male	76.3
female	23.7
Age, median (range) [years]	66.5 (37–89)
BMI, median (range) [kg/m^2^]	25.65 (19.23–36.72)
Hepatic malignancy [%]	
HCC	76.3
Liver metastasis (CRC)	10.5
Liver metastasis (gastric cancer)	2.6
Liver metastasis (pancreatic)	5.3
Liver metastasis (CCA)	5.3
Tumor size, median (range) [cm]	2.8 (1.2–12.9)
Cause of HCC [%]	
alcoholic	31.0
HCV	20.7
HBV	6.9
cryptogenic	20.7
others (e.g. NASH)	19.7
Child Pugh stage of cirrhosis (HCC only) [%]	
Child Pugh A	
Child Pugh B	
OR to TACE therapy [%]	
yes	45.2
no	54.8
Deceased during follow-up [%]	
yes	73.0
no	27.0
Concentration of EV, median and range [p/mL]	1.55x10^11^ (0.19–4.5)
Size of EV, median and range [nm]	142.3 (110.9–162.5)

TACE, transarterial chemoembolization; BMI, body-mass-index; HCC, Hepatocellular carcinoma; CRC, Colorectal carcinoma; CCA, Cholangiocarcinoma; HCV, hepatitis C virus; HBV, hepatitis B virus; NASH, non-alcoholic steatohepatitis; CHILD, Pugh-Child score; OR, objective response; EV, extracellular vesicles; p, particles

### Concentration and size of extracellular vesicles in patients with primary and secondary liver cancer

**Fig 1:**
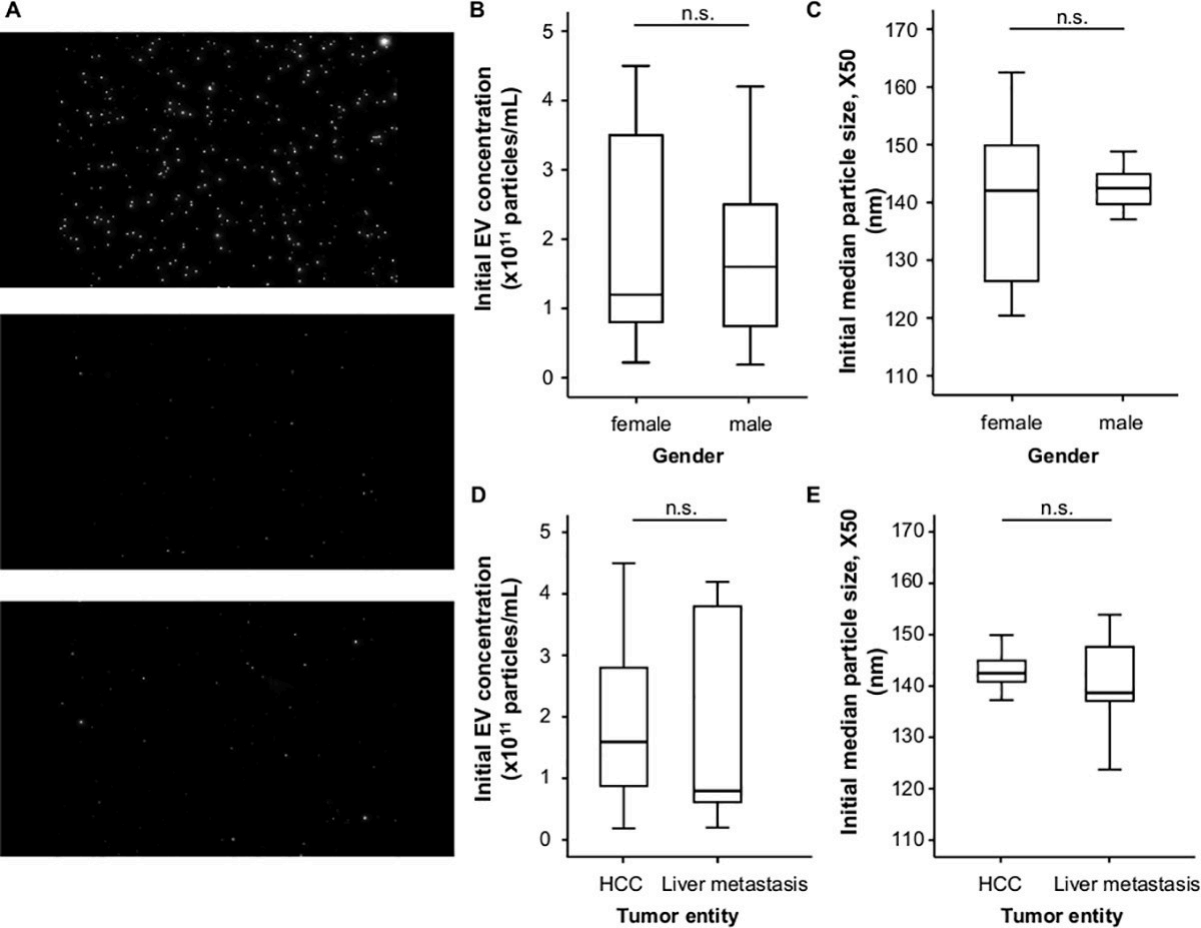
EVS concentration and size does not differ between female and male patients and between HCC and liver metastases before TACE. (A, up) NTA camera detection of a 1:250000 standard solution at the beginning of measurements. (A, middle) NTA camera view of a sample at baseline. (A, bottom) NTA camera view of a sample at d1 after TACE. (B) Initial EVs concentrations and (C) initial median particles sizes (X50) are comparable between female and male patients. (D) Initial EVS concentrations do not differ between patients with HCC and patients with liver metastases. (E) Initial median particle size (X50) does not differ between patients with HCC compared to patients with liver metastases.

**Fig 2:**
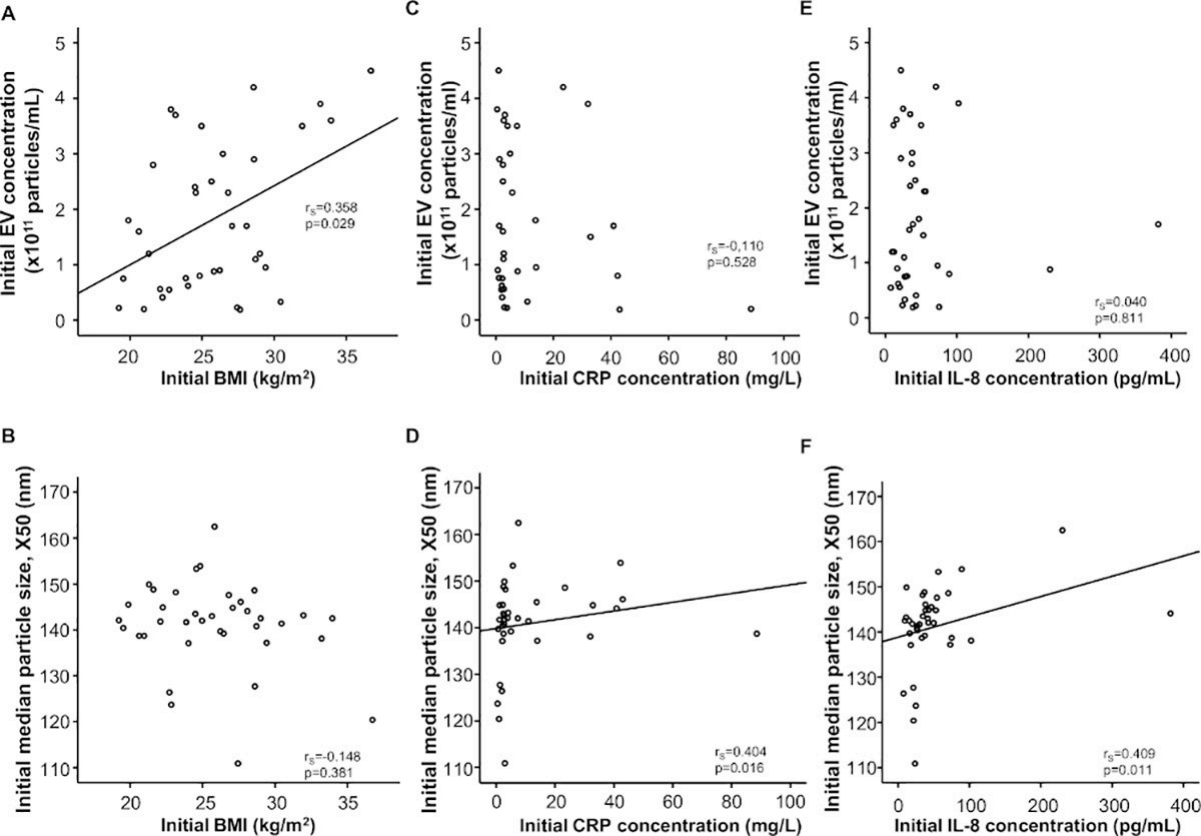
EVS size (X50) positively correlates with markers of inflammation before TACE. (A) Initial BMI significantly correlates with initial EVs concentration. (B) BMI does not correlate with initial median particle size (X50). (C) Initial EVs concentration does not correlate with CRP-concentration. (D) Initial CRP-concentration significantly correlates with initial median particle size (X50). (E) Initial EVS concentration does not correlate with IL-8-concentration. (F) Initial IL-concentration significantly correlates with initial median particle size (X50).

The median circulating EVs concentration of our TACE cohort was 1.55 x10^11^ (range: 0.19–4.5x10^11^ particles/mL) while the median EVs size (X50) was 142.3 nm and ranged from 110.9 to 162.5 nm, as measured by Nanoparticle Tracking Analysis (NTA; Fig 1A). In order to gain first insights into the regulation of EVs concentrations and size in patients with primary or secondary liver cancer, we compared these parameters between different subgroups of our cohort. Here, we did not observe a significant difference between male or female patients (Fig 1B and 1C) or patients with HCC or liver metastases (Fig 1D and 1E). Interestingly, we observed a significant positive correlation between initial circulating EVs concentrations and the patients’ body-mass index (BMI, r_S_: 0.358, p = 0.029, Fig 2A), while the EVs size was not affected by the BMI (r_S_: -0,148, p = 0.381, Fig 2B). EVS concentrations and size did not significantly correlate with patients’ age.

We next performed extensive correlation analyses between EVs concentrations/size and numerous lab parameters of organ dysfunction. We observed a significant positive correlation between EVs size (X50) and parameters of systemic inflammation. While there was no significant correlation between C-reactive protein concentration and initial circulating EVs concentration (r_S_: -0,110, p = 0.528, Fig 2B), EVs size (X50) significantly correlated with C-reactive protein (r_S_: 0.404, p = 0.016, Fig 2D). While Il-8 concentration and initial circulating EVs concentration also did not significantly correlate (r_S_: 0.040, p = 0.528, Fig 2E), EVs size (X50) correlated significantly with IL-8 concentrations (r_S_: 0.409, p = 0.011, Fig 2F). Moreover, EVs concentration correlated with IL-16, but did not reach significance (r_S_: -0.320, p = 0.051). In addition, we observed no correlation between EVs concentration or size with MELD score, creatinine, sodium or potassium.

### Extracellular vesicle size and concentration and tumor response to TACE therapy

**Fig 3:**
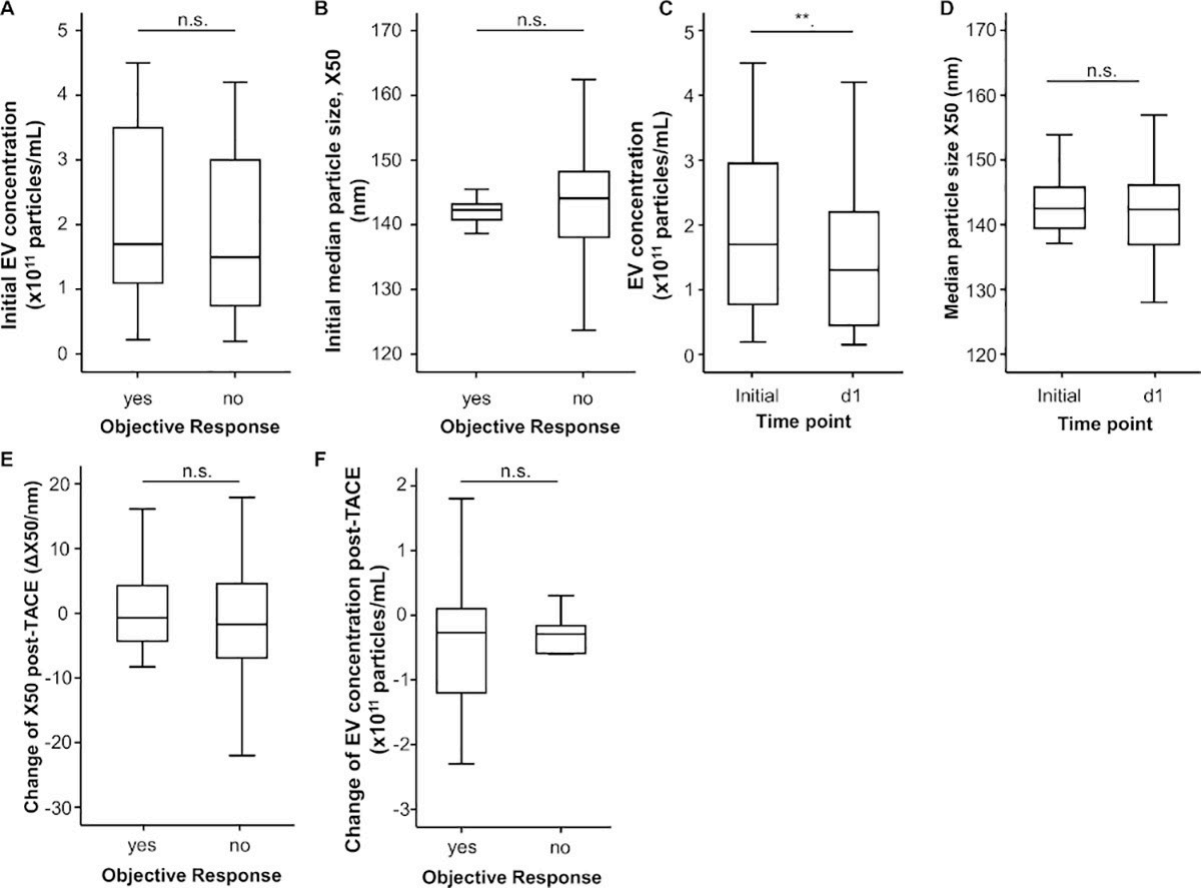
Particle size (X50) and particle concentration do not differ in patients with and without objective response (OR) after TACE. (A and B) Initial EVs concentrations and initial median particle sizes do not differ between patients with and without OR after TACE. (C) EVs concentration is significantly reduced in patients at day 1 compared to baseline, whereas (D) median particle size (X50) does not differ between baseline and day 1 after TACE. (E) The differences of X50 (ΔX50) and (F) of EVs concentrations pre-/post-TACE do not differ between patients with and without OR.

In a next step, we hypothesized that EVs concentrations or size before TACE might be predictive for the patients’ individual tumor response to TACE. However, we observed comparable baseline EVs concentrations between patients who showed an OR to TACE and non-responders (Fig 3A). In addition, the size of EVs before TACE were comparable between OR and non-OR patients (Fig 3B). In line, univariate binary logistic regression analyses for the prediction of an OR to TACE therapy revealed that neither initial EVs concentrations nor the EVs size were a suitable predictive marker for an OR (OR_conc_: 1.000, 95%CI: 1.000–1.000, p = 0.620; OR_size_: 1.025, 95%CI: 0.938–1.120, p = 0.588). In a last step, we evaluated whether the individual dynamic of EVs concentrations/size before and one day after TACE might represent a predictive marker for TACE response. The EVs concentrations were significantly lower at day 1 after TACE compared to the respective baseline values (p = 0.004, Fig 3C). Although the EVs sizes were similar between the two time-points (Fig 3D), the delta of both parameters was comparable between OR and non-OR patients (Fig 3E and 3F).

### Baseline size of extracellular vesicles are a prognostic marker for overall survival following TACE therapy

**Fig 4:**
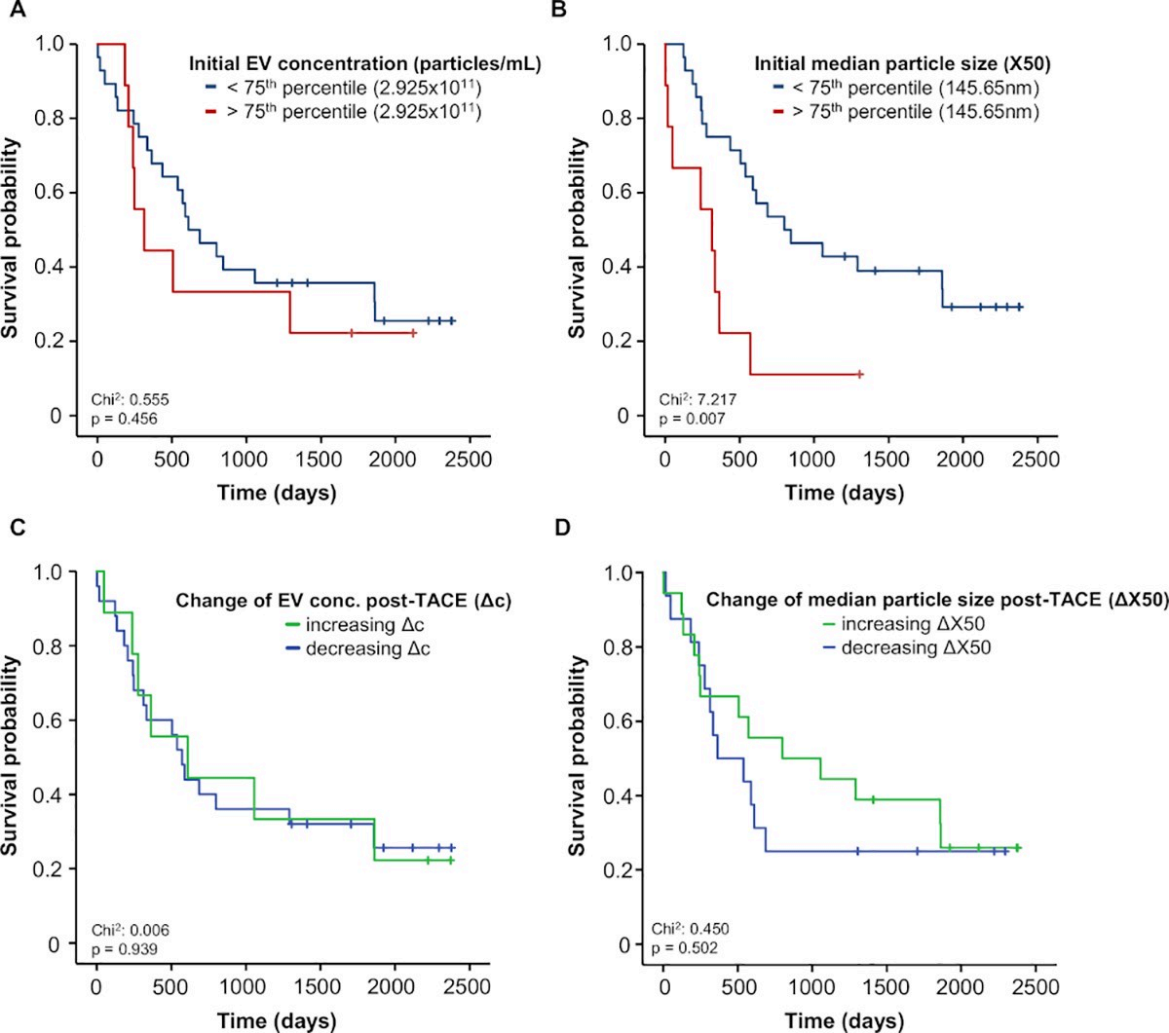
Elevated baseline median particle size predicts an unfavorable outcome after TACE. (A) Initial EVS concentration does not predict overall survival in patients with primary or secondary hepatic cancer. (B) Patients with an initial median particle size (X50) above the 75^th^ percentile (145,65nm) shows a significantly impaired post-interventional survival. (C+D) Increases and decreases of EVs concentrations and median particle sizes (X50) between baseline and day 1 after TACE do not predict overall survival in patients with primary or secondary hepatic cancer.

Finally, we aimed at evaluating whether concentrations or size of EVs might represent a prognostic marker of overall survival (OS) in liver cancer patients undergoing TACE. In order to test this hypothesis, we compared OS of patients with high/large EVs concentrations/size (in the 4^th^ quartile) to patients with EVs concentrations/size in the 1^st^ to 3^rd^ quartile. While, we did not observe a significantly impaired OS in patients with high or low EVs concentrations (Fig 4A), patients with an EVs size within the 4^th^ quartile had a significantly impaired OS compared to patients with smaller EVs (Fig 4B). The median OS of the EVs size high group was only 314 days compared to 799 days in liver cancer patients from the EVs size low group. In line, univariate Cox-regression analyses revealed that patients’ baseline EVs size, but not EVs concentrations, were a significant prognostic factor for OS (Table 2).

**Table 2:** Uni- and multivariate Cox-regression analysis for the prediction of overall survival after TACE.

	univariate Cox-regression		multivariate Cox-regression	
Parameter	p-value	Hazard-Ratio (95% CI)	p-value	Hazard-Ratio (95% CI)
EVS concentrations	0.775	1.000 (1.000–1.000)		
EVS size	0.010	1.089 (1.021–1.162)	0.014	1.097 (1.018–1.181)
Age	0.477	1.014 (0.976–1.053)		
Sex	0.320	0.609 (0.229–1.619)		
BMI	0.286	0.950 (0.864–1.044)		
Size of target lesion	0.514	1.004 (0.992–1.017)		
Potassium	0.280	1.550 (0.700–3.434)		
Leukocyte count	0.139	1.125 (0.962–1.314)	0.564	1.068 (0.854–1.337)
Bilirubin	0.786	1.116 (0.504–2.473)		
Creatinine	0.109	1.556 (0.906–2.674)	0.416	1.259 (0.723–2.191)
CRP	0.020	1.021 (1.003–1.039)	0.404	1.012 (0.984–1.041)

EV: Extracellular vesicles, BMI: Body-Mass-Index, CRP: C-reactive protein

To identify potential confounders, we included parameters with a p<0.250 into multivariate testing. Importantly, the prognostic relevance of the EVs size was independent of several clinicopathological parameters in multivariate Cox-regression analysis (HR: 1.097, 95%CI: 1.018–1.181, p = 0.014, Table 2). In a last step, we evaluated whether the individual course of EVs concentrations/size before and at day 1 might have an impact on patients’ OS. However, patients with increasing or decreasing EVs concentrations one day after TACE showed a comparable OS (Fig 4C). In terms of EVs size, we observed trend towards an impaired OS in patients with further increasing EVs size after TACE but statistical significance was not reached (Fig 4D).

## Discussion

Recently, the introduction of the concept of multimodal treatment has changed our view of how to treat primary and secondary liver cancers. Besides the introduction of highly active chemotherapies, innovative locally ablative techniques such as the transarterial chemoembolization have considerably improved patients´ prognosis. Nevertheless, it has become clear that only a part of patients display response to these treatments while other patients only suffer from treatment side effect without showing clinical benefit [3, 16]. In this study, we analyzed the prognostic and predictive potential of measurements of extracellular vesicle concentrations and sizes in patients that were allocated to TACE for different tumor entities, with HCC representing the most important etiology. We demonstrate that pre-interventional sizes but not concentrations of EVs have a prognostic potential in the context of TACE since patients who displayed EVs diameters (as measured by X50) above 145.65nm exhibited a significantly impaired prognosis compared to all other patients.

Deciding which patients should receive TACE and which should not, is a major clinical challenge. Current stratification systems are largely based on values that reflect general condition or tumor stage but do not adequately take tumor biology into account. Against this background, our data on a prognostic role of EVs could provide valuable additional information, especially when integrated into existing clinical scoring systems. It is important to underline, that there is great interest to measure the concentration of EVs in a sample together with their size distribution. For example, EV size is used to infer the type of EVs (e.g., exosomes, microvesicles and apoptotic bodies), although the relation between EV size and EV type is less defined than suggested. A number of studies have previously reported that the size and concentration of EVs vary in different stages of different cancer types, suggesting that these parameters are potentially useful for clinical diagnostics [18, 19]. More recently alterations in EVs concentrations and characteristics (e.g., cargo) were recognized as novel biomarkers in the context of malignant diseases. Different studies have reported alterations in mean vesicle size and concentrations in different liver diseases including non-alcoholic and alcoholic steatohepatitis, chronic viral hepatitis B and C infections, cirrhosis, primary sclerosing cholangitis and acute liver failure [20]. Here we demonstrate a potential role of mean EVs size as a prognostic marker in patients receiving TACE for primary and secondary liver cancer. Notably, our data are supported by previous studies showing that EVs predict HCC recurrence after liver resection and that circulating extracellular vesicle RNA expression can also predict overall survival in HCC patients [21–24]. Moreover, higher levels of RAB11A RNA in small extracellular vesicles in the serum were associated with lower relapse-free survival among 60 patients with mainly early HCC in multivariate analysis [25]. Besides predicting patient’s prognosis several other end-points including response to treatment were tested. However, at least in our cohort alterations in mean EVs size or concentration did not reflect response to TACE. This discrepancy clearly raises the question which mechanism are driving alterations in EVs characteristics under physiological and pathophysiological conditions in human. It was recently demonstrated that in NASH, an inflammatory liver disease, EV-mediated intercellular and interorgan crosstalk represents central events in the progression of the disease. Moreover, besides liver diseases alterations in EVs were recognized as key events in many other inflammatory diseases such as autoimmune disorders, atherosclerosis [26, 27] and bacterial as well as viral infection [10, 28]. In line, to these findings as well as on the prognostic role of EVs mean sizes, but not concentrations, we demonstrate a statistically significant correlation between EVs size and serum levels of CRP as well as IL-8. Thus, our data support the hypothesis that alterations in EVs mean sizes reflect inflammatory processes that potentially negatively affect the patients´ prognosis.

Extracellular vesicles are ubiquitous masters of intercellular communication, being present in body-fluids and fractions thereof, such as serum or plasma, meaning that their associated cargo not only reflects the (patho)physiologic status of the secreting cells but it may also influence so called recipient cells [29]. In the context of inflammation it was recently demonstrated that miRNA- let-7e-5p, contained in EVs derived from hepatocytes enhances lipid accumulation in adipocytes [30]. Moreover, EV-miR-192-5p from hepatocytes activate macrophages and increase IL-6 and TNF-α expression, promoting inflammation in NAFLD, summarized in [31]. Thus, beyond demonstrating a potential role of EV as biomarkers in patients receiving TACE our data also provide pathophysiological insights into different processes determining the prognosis of these patients. In addition, the cause for EV concentration decrease after TACE, as shown in this study, remains to be investigated in future studies, i.e. whether they derive from cancer cells and with view to their function.

In summary, the data included in this study support the conclusion that the mean size of EVs, but not their concentrations, may function as an independent marker to discriminate between patients with favorable and unfavorable prognosis in Kaplan-Meier and Cox-regression analyses. Since our study included both patients with primary and secondary hepatic malignancies, our data argue for an entity independent prognostic value of EVs mean size in patients receiving TACE. We acknowledge the limitations of our analyses. Most importantly, the number of patients included into our study is small with only 38 analyzed patients. Moreover, all patients were included at one center. Finally, our study was not restricted to one tumor type, since both patients with primary and secondary hepatic malignancies were included. Therefore, larger clinical trials featuring a prospective multicentric design are needed before a use of EVs concentrations and sizes in clinical routine should be considered. Nevertheless, ours is the first study to analyze these parameters in patients receiving TACE for primary or secondary liver malignancies and we provide evidence for a previously unrecognized role of EVs size as a biomarker in this context.

## Supporting information

S1 Fig Initial median particles size (X50) does not correlate with initial EVS concentrations or size of target lesion. (A) Initial median particle size (X50) does neither correlate with initial EVs concentration nor (B) with size of target lesion.
(TIFF)
