# Supplementary figures and images for: Enlarged extracellular vesicles are a negative prognostic factor in patients undergoing TACE for primary or secondary liver cancer–a case series
Source: PLoS One. 2021 Aug 18;16(8):e0255983. doi: 10.1371/journal.pone.0255983 (PMC8372935; doi:10.1371/journal.pone.0255983)

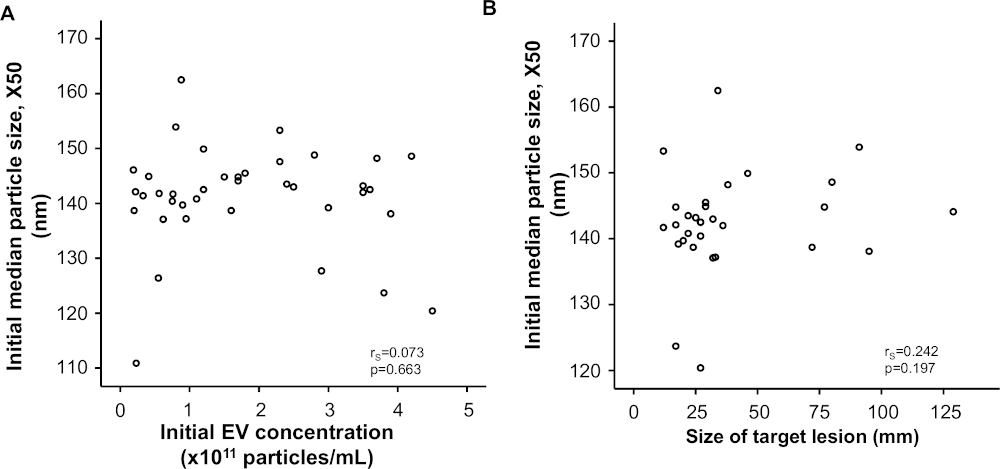

Supplement: S1 Fig — (A) Initial median particle size (X50) does neither correlate with initial EVs concentration nor (B) with size of target lesion. (TIFF) [file pone.0255983.s001.tiff]
